# Avian Conservation Areas as a Proxy for Contaminated Soil Remediation

**DOI:** 10.3390/ijerph120708312

**Published:** 2015-07-17

**Authors:** Wei-Chih Lin, Yu-Pin Lin, Johnathen Anthony, Tsun-Su Ding

**Affiliations:** 1 Department of Bioenvironmental Systems Engineering, National Taiwan University, No. 1, Sec. 4, Roosevelt Road, Taipei 10617, Taiwan; E-Mails: b97602046@ntu.edu.tw (W.-C.L.); moose_me_dowg@hotmail.com (J.A.); 2 School of Forestry and Resource Conservation, National Taiwan University, No. 1, Sec. 4, Roosevelt Road, Taipei 10617, Taiwan; E-Mail: ding@ntu.edu.tw

**Keywords:** remediation priority, multiple heavy metals, pollution, biological conservation, robust decision, geostatistics

## Abstract

Remediation prioritization frequently falls short of systematically evaluating the underlying ecological value of different sites. This study presents a novel approach to delineating sites that are both contaminated by any of eight heavy metals and have high habitat value to high-priority species. The conservation priority of each planning site herein was based on the projected distributions of eight protected bird species, simulated using 900 outputs of species distribution models (SDMs) and the subsequent application of a systematic conservation tool. The distributions of heavy metal concentrations were generated using a geostatistical joint-simulation approach. The uncertainties in the heavy metal distributions were quantified in terms of variability among 1000 realization sets. Finally, a novel remediation decision-making approach was presented for delineating contaminated sites in need of remediation based on the spatial uncertainties of multiple realizations and the priorities of conservation areas. The results thus obtained demonstrate that up to 42% of areas of high conservation priority are also contaminated by one or more of the heavy metal contaminants of interest. Moreover, as the proportion of the land for proposed remediated increased, the projected area of the pollution-free habitat also increased. Overall uncertainty, in terms of the false positive contamination rate, also increased. These results indicate that the proposed decision-making approach successfully accounted for the intrinsic trade-offs among a high number of pollution-free habitats, low false positive rates and robustness of expected decision outcomes.

## 1. Introduction

Heavy metals are common by-products of, and are mobilized by, human activities and are therefore prevalent in many natural habitats such as forests, rivers, lakes and oceans [1,2,3,4,5]. Unfortunately, contaminant remediation projects sometimes fail to consider the ecological importance of specific sites, which is crucial in areas where high population densities and rapid industrialization restricts prospective conservation areas, such as low-land Taiwan [6,7]. Since remediation efforts which consider ecological value depend on the integration of many models, robust decision making tools must be developed to evaluate the uncertainty of estimates [6] and to manage environmental contamination in a cost-effective manner [8], based on both conservation priorities and the contamination status of given sites.

While the contamination of soil by heavy metals detrimentally affects most species, certain species are particularly vulnerable to environmental contaminants; avian populations, for instance, are extremely sensitive to heavy metals, so much so that bird scarcity has often been used as a bioindicator of heavy metal pollution [9,10]. As such they could be threatened as their ranges are slowly reduced to conservation areas that are possibly contaminated, particularly in densely populated countries where land is at a premium. According to previous studies, heavy metal pollution has immediate adverse health effects on birds [9,11,12,13,14], and is known to cause a severe decline in avian reproduction [15]. Heavy metal pollution from industrial activities that accumulates in prey can lead to the complete extinction of some species [15,16] as it has significant long-term, adverse effects on most species [9,17] and particularly adverse effects on bird reproduction [17,18]. Moreover, a combination of debilitation by heavy metals, associated interference of calcium metabolism and increased methylmercury concentrations, can result in the formation of poor-quality egg shells, and increased egg and nestling mortality [11,19,20,21]. Ruuskanen *et al.* identified significant As, Cd, Cr, Cu, Ni, Pb, Zn, Se, Sr, and Ca, (but not Cu) metal concentration variations in the eggshells of focal species [10]. Accordingly, given the possible adverse effects of heavy metal contamination on birds, the delineation of protect areas should prioritize either the avoidance or the removal of heavy metal pollution.

Modern initiates for selecting reserve areas frequently depend on computational algorithms, known as systematic conservation planning (SCP) platforms, such as zonation [22]. Zonation generates a hierarchy of conservation priorities based on biodiversity, cost, and minimization of marginal loss to each habitat in a planning region [22]. The process involves the iterative removal of cells that least reduce the conservational value of the remaining reserve network [22]. Recently, zonation has been used in conservation planning for bird species [7,23,24]. Unfortunately, most systematic conservation planning strategies do not quantitatively consider environmental pollution, such as heavy metals, which can adversely influence protected species and are highly prevalent in densely populated areas such as Taiwan [10,12,16].

In soil science, single variable simulation methods may be ineffective for generating spatial concentration estimates owing to their inability both to incorporate correlations between variables such as multiple heavy metal concentrations [25] and to correctly assess the multivariate grade risk [26]. The method of minimum/maximum autocorrelation factors (MAF) assumes that the semivariogram function of each attribute can be modelled by a bi-structural linear model based on coregionalization, and transforms measured original data into non-orthogonal factors with weak spatial correlations by diagonalizing a pair of symmetrical coregionalization matrices [27]. Tichavsky and Yeredor presented a more general approach to approximate joint diagonalization (AJD), called Uniformly Weighted Exhaustive Diagonalization with Gauss iterations (U-WEDGE) [28]. The advantage of U-WEDGE is that it makes no assumption about the structure of the semivariogram function. The resultant realizations of multivariate attributes, simulated by AJD, have been found to be comparable to those generated by a full joint-simulation [27,29,30].

In this study the impacts of heavy metal pollution on potential conservation areas in low-land Taiwan was considered, furthermore, the benefits of remediating these areas were also evaluated. Suitability of conservation areas were considered in terms of how well they cover the distributions, *i.e.*, preferences, of all focal species, while remediation priority was based on the pollution and conservation status of each site. Since all of the above heavy metal simulations and species distribution projections involve a high degree of uncertainty, a systematic approach was taken to provide decision-makers with a structured means of making robust decisions under uncertainties that arise from multiple sources [8,31,32,33]. The remediation-driven decision-making approach developed here specifies the effectiveness of different decisions, *i.e.*, total area remediated, in terms of not only the projected resultant habitat quality, but also how certain we are of meeting specific conservation objectives.

## 2. Methods and Materials

**Figure 1:**
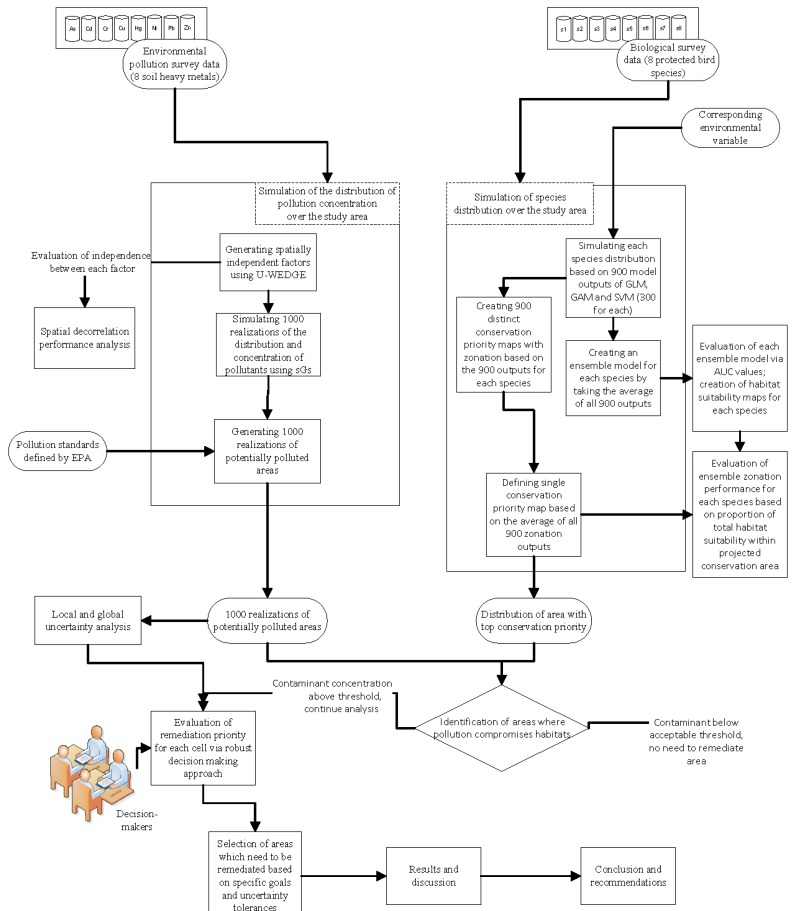
Flowchart of the approach of this study.

The flowchart of the approach used in this study is illustrated in Figure 1. To evaluate the impacts of heavy metal-contaminated soils on focal species within the proposed conservation areas, the concentrations of eight heavy metals were projected across the study area. The analysis of soil contaminants comprised the following steps. First, the U-WEDGE method was used to transform the concentrations of the eight metals into eight spatially uncorrelated factors to perform multivariate simulations without the need to consider the interactions among the eight metals. The normalized uncorrelated factors were then fed into sequential Gaussian simulation (sGs), which generated 1000 realizations of possible soil heavy metal distributions, instead of simply projecting a single estimate. sGs therefore allowed for more realistic uncertainty analysis. The local and spatial (global) uncertainties of areas that require monitoring or remediation were then analyzed. In terms of species distributions, a total of eight bird species, seven with “rare and valuable” protection status [34], were considered. The analysis of the distribution of species and the selection of conservation areas involved two ensemble modeling techniques that considered both individual species habitat preferences and the conservation needs of all species of interest. The first integrated 300 outputs of three species distribution models (SDM) into a single ensemble SDM. Zonation was then applied to the 900 outputs of the SDMs to generate 900 conservation priority maps for each species. The average of these conservation priority realizations was used as the ensemble conservation output that took into account the distribution of all eight species. The ensemble zonation output ultimately determined the ecological value, or from the point of view of policy makers the top conservation priority, *i.e.*, ecological value in descending order, of each cell (planning unit). Finally, remediation priorities were obtained from the ecological values of focal bird species, the heavy metal distribution maps, as well as the level of uncertainties in heavy metal pollution projections.

### 2.1. Study Area and Data

**Figure 2:**
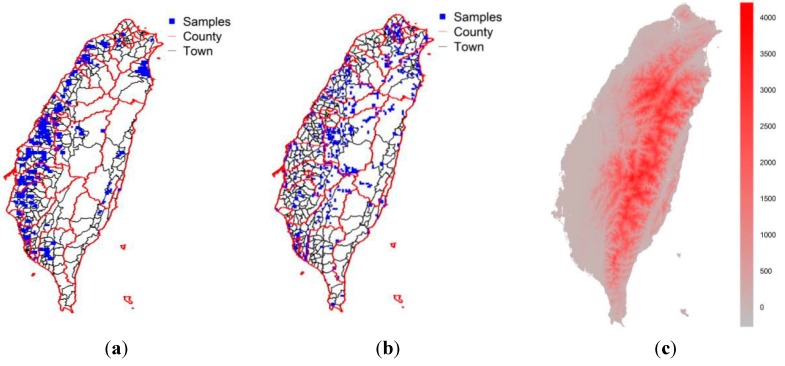
(**a**) Sites where soil samples were obtained in the second step of heavy metal sampling; (**b**) sites where birds were sampled (**c**) digital elevation map.

Taiwan is a subtropical island with an area of 36,000 km^2^ (Figure 2). The eight bird species that were considered in this study are *Accipiter trivirgatus*, *Milvus migrans*, *Hydrophasianus chirurgus*, *Rostratula benghalensis*, *Glareola maldirarus*, *Otus lettia*, *Garrulax Taewanus*, and *Acridotheres cristatellu* (Table S1 and photographs in Figure S1). The Taiwanese protection level of the first seven of these species is “rare and valuable” while that of the last species is “conservation-deserving” [34]. Most of the habitats of these species are in low-altitude areas, which are more likely to be contaminated by heavy metal pollution on account of human activities. The species were selected precisely because their conservation protection level in most cases was “rare and valuable” and their habitats were predominantly in low-altitude areas (Table S1), so they were liable to be exposed to heavy metal pollution. Table S1 presents background information concerning the eight species.

Heavy metal concetrations were obtained from a nationwide survey that was performed by the Environmental Protection Administration (EPA) of Taiwan and involved 2183 soil samples based on a hierarchical sampling structure. In the first step, there were 897 soil samples collected at a resolution of 1600 ha across of Taiwan. Finally, 2183 sites were then sampled across sample units of 100 ha within sites identified as potentially contaminated during the first step. As can be seen, the majority of the sites considered in the second phase of the study were located on the highly industrialized west coast of Taiwan. The soil concentrations of arsenic (As), cadmium (Cd), chromium (Cr), copper (Cu), mercury (Hg), nickel (Ni), lead (Pb), and zinc (Zn) at depths of 0–15 cm were considered. The EPA classifies the concentrations of soil heavy metals into five classes (Table S2, the Supplementary Section 2). Concentrations in the fourth and fifth ranges require intensive monitoring and consideration of remediation. In this study, areas with concentrations within or above that of the fourth range are regarded as polluted.

### 2.2. Systematic Conservation Planning Using Zonation and Evaluation of Identification of Conservation Areas

The individual distributions of the eight bird species were ensemble-modeled based on 300 runs of three species distribution models (SDMs), which were the general linear model (GLM), the general additive model (GAM) and the support vector machine (SVM) (Supplementary Table S5). The models were constructed using both bird presence-absence data and corresponding background environmental variables [35] (Supplementary Section 4). Lee *et al.* and Koh *et al.* elucidated most of these background environmental variables [36,37]. To model the distribution of bird species in Taiwan, Ko *et al.* used over 80 environmental variables, classified into four categories, which were topography/geography, climate, vegetation/land use, and human activities, and included all of the background environmental variables considered herein [35]. Ko *et al.* indicated that, among these environmental variables, elevation, NDVI, temperature, road density, and forest density determined the distributions of species via the location of biodiversity hotspots [35]. With respect to species distribution modeling, the data for the eight species were randomly separated into a training set, which comprised 80% of the data, and a validation set, which comprised the remaining 20%. Moreover, based on the latter 20%, the area under the receiver operating characteristic curve (AUC) was used to characterize the capacity of the ensemble SDM models to distinguish presence-absence records from background data [7]. Based on the above 900 SDM outputs for each species (7200 total), 900 conservation area realizations were generated and the average of these determined the ultimate conservation priority of each area.

From the average ensemble zonation output, the areas with the top 5%, 10%, 20% and 30% conservation priorities were selected as protected areas. The selection of protected areas was based on not only conservation targets but also pseudo targets, which represent the likelihood of the presence of the target species based on distribution predictions obtained from each SDM output. In zonation with the additive benefit function, cells are removed iteratively in a manner that minimizes habitat loss: cells i that have the smallest $\delta_{i}$ are removed, maximizing the representation of the species in question, while considering landscape metrics, or, more specifically, maximizing core areas [22]:

(1) $$\delta_{i}=\underset{j}{\sum }w_{j}\left[R_{j}\left(S\right)-R_{j}\left(S-i\right)\right]$$

where $w_{j}$ is the weight (or priority) of species *j.* In this study, the eight species are equally weighted. $R_{j}\left(S\right)$ is the representation of species j in the remaining set of sites S, and (S−i) is the set of remaining cells minus cell i. Initially, when the full landscape is considered, $R_{j}\left(S\right)=1$ for all species; as cell removal proceeds, $R_{j}\left(S\right)$ decreases.

### 2.3. Modeling of Heavy Metal Distribution Using U-WEDGE and Sequential Gaussian Simulation

U-WEDGE was used for decorrelation in this study. A decorrelation matrix V, which is a diagonal matrix for each ${\text{M}}_{\text{j}}$ in ${\text{VM}}_{\text{j}}{\text{V}}^{\text{T}}$ was determined, where ${\text{M}}_{\text{j}}$ are symmetrical K×K spatial correlation matrices between metals in each lagj=1,2,…,J. To obtain the decorrelation matrix V, the following equation with fixed point iterations is minimized [27,28]:

(2) $${\text{C}}_{\text{LS}}\left(\text{V},\text{A}\right)=\overset{\text{J}}{\underset{\text{j}=1}{\sum }}∥{\text{VM}}_{\text{j}}{\text{V}}^{\text{T}}-{\text{AD}}_{\text{j},\text{V}}{\text{A}}^{\text{T}}{∥}_{\text{F}}^{2}$$

where $∥\cdot {∥}_{\text{F}}$ is the Frobenius norm, and ${\text{D}}_{\text{j},\text{V}}$ is the series of diagonal matrices that are derived from ${\mathrm{VM}}_{\text{j}}{\text{V}}^{\text{T}}$, which can be expressed as $\text{\{diag}\left({\text{VM}}_{\text{j}}{\text{V}}^{\text{T}}\right)|\text{j}=1,2,\ldots ,\text{J}\}$. The matrices A and V are known as the mixing and demixing matrices, respectively.

In this work, the target matrices $\left\{{\text{M}}_{1},{\text{M}}_{2}\ldots {\text{M}}_{J}\right\}$ are cross-correlogram matrices between pairs of the eight heavy metal concentrations at J separations. The factors $F_{\text{U}-\text{WEDGE}}$ that were derived by U-WEDGE algorithm are written as

(3) $$F_{\text{U}-\text{WEDGE}}=\text{V}\times Z$$

where $Z={\left[{\text{Z}}_{1},{\text{Z}}_{2},\ldots ,{\text{Z}}_{8}\right]}^{\text{T}}$, ${\text{Z}}_{1},{\text{Z}}_{2},\ldots ,{\text{Z}}_{8}$ are the concentrations of the eight heavy metal contaminants of the soil.

The spatial correlation between the factors $F_{\text{U}-\text{WEDGE}}$ can be evaluated using various indices, which are absolute deviation from diagonality ς(h), relative deviation from diagonality τ(h), and spatial diagonalization efficiency κ(h) [27]. Perfect spatial decorrelation occurs when ς(h)=τ(h)=0 and κ(h)=1. for all lag h. Xie suggested that the performance of the decorrelation method is acceptable if κ(h)≥0.9. [38]. The global indices of the spatial correlation that remains after decorrelation are $\bar{\varsigma }$, $\bar{\tau }$, and $\bar{\kappa }$, calculated over J lags, which can be obtained as the averages of ς(h), τ(h) and κ(h) over J lags, respectively.

The decorrelated outputs from U-WEDGE underwent normalization [39], and were then used as inputs in the sGs, which generated 1000 realizations of heavy metal distributions. Supplementary Section 5 presents the details of sGs, as well as the basic requirement specifics of normal score transformation to ensure the normality of data distribution [40].

### 2.4. Analysis of Local and Spatial Uncertainties of Heavy Metal Concentrations

The local uncertainty $P_{l}\left(x^{\prime }\right)$ at location $x^{\prime }$ is defined as follows [41]:

(4) $$P_{l}\left(x^{\prime }\right)=Prob\left[I\left(x^{\prime }\right)=1\right]=v\left(x^{\prime }\right)/L$$

where $I\left(x^{\prime }\right)=1$ when $x^{\prime }$ is a potentially contaminated cell or $I\left(x^{\prime }\right)=0$ when it is not; L is the total number of realizations, and $v\left(x^{\prime }\right)$ is the number of L realizations in which $x^{\prime }$ is a potentially contaminated cell. In this case, 1000 realizations (L = 1000) were used to generate reliable results used in the uncertainty analysis [42].

The joint probability ($P_{j}$, global uncertainty) of the 1000 realizations at m locations $\left({\text{u}}_{1},{\text{u}}_{2},{\text{u}}_{3},\ldots ,{\text{u}}_{\text{m}}\right)$, was defined in terms of a sequence of increasing cut-off values, herein referred to as critical proportions [7,43]:

(5) $$P_{j}={\text{n}}_{\text{p}}\left({\text{u}}_{1},{\text{u}}_{2},{\text{u}}_{3},\ldots ,{\text{u}}_{\text{m}}\right)/\text{L}$$

where L is the total number of realizations; ${\text{n}}_{\text{p}}\left({\text{u}}_{1},{\text{u}}_{2},{\text{u}}_{3},\ldots ,{\text{u}}_{\text{m}}\right)$ is the number of the L realizations in which the total projected polluted areas exceeded the predefined critical proportion; m is the number of cells in which the proportion of the study area that is estimated to be polluted exceeded the preset critical proportion for polluted sites; *np* is the number of realizations (out of L) in which the projected contamination area exceeds the critical proportion for the polluted sites.

### 2.5. Decision-Making Concerning Remediation 

The effectiveness of decisions was evaluated by both minimizing the false positive identification of heavy metal contaminants, and by maximizing the ecological value, given environmental pollution (EVEPI). The false positive rate is calculated by dividing the number of non-polluted areas that are wrongly classified as contaminated by the total number of non-polluted areas. The index of ecological value based on environmental pollution, herein referred to as the ecological value requirement or pollution-free habitat suitability, is defined as,

𝐸𝑉𝐸𝑃𝐼𝑐=𝐸𝑉𝐼𝑐(1−𝐸𝑃𝑐)(6)

where $EVI_{c}$ is the ecological value, or conservation priority in ascending order, of cell c, and is a value between zero and one, which is obtained from ensemble zonation. $EP_{c}$ represents the contamination status of cell c; if $EP_{c}=1$, then at least one of the concentration thresholds for any of the heavy metals has been exceeded in cell c and it is therefore unsuitable for species habitation.

Owing to the uncertainties associated with contamination, each remediation strategy s corresponds to varying false positive rates, ${\tilde{F}}_{s}$, and ecological value requirements, ${\tilde{EVEPI}}_{s}$, under varying realizations of heavy metal contamination. In the proposed remediation decision-making approach, the robustness function, $\hat{\text{r}}\left(H\right)$, is defined in terms of the number of realizations for which the user-defined $F_{s}$, and *EVEPI,* are obtained. These reflect the average of 900 realizations of conservation priority and the proportion of 8000 heavy metal realizations of zonation and sGs projections, respectively, which meet the predefined objectives. H is a vector that contains the various cut-off values for total heavy metal area remediation scenarios. Decision-makers can use the robustness function to evaluate the reliability of the setting for each cut-off value for heavy metal remediation.

## 3. Results

### 3.1. Species Distribution and Protected Areas

**Figure 3:**
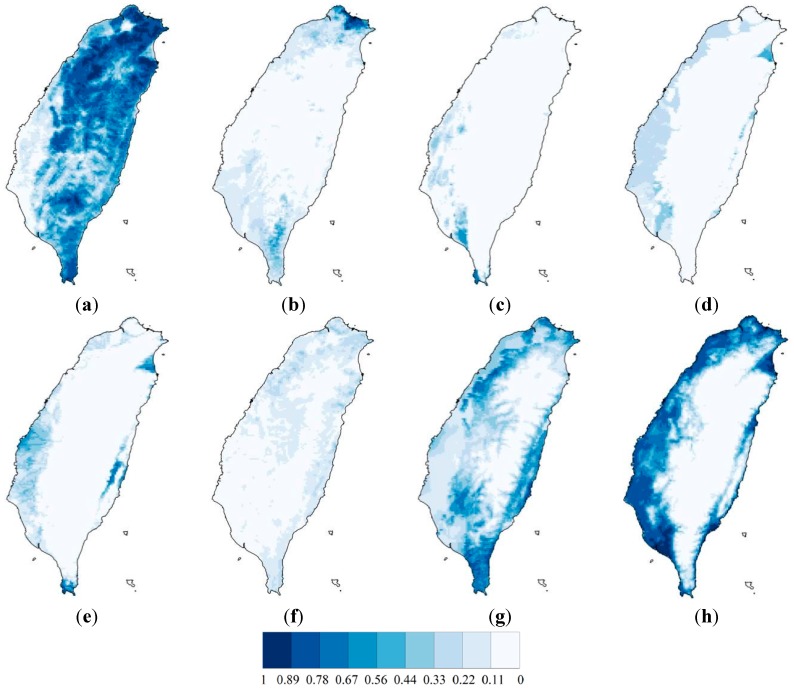
The average habitat suitability index projected by 900 outputs of general linear model, general additive model and supporting vector machine for (**a**) *A. trivirgatus*; (**b**) *M. migrans*; (**c**) *H. chirurgus*; (**d**) *R. benghalensis*; (**e**) *G. maldirarus*; (**f**) *O. lettia*; (**g**) *G. Taewanus*and; (**h**) *A. cristatellu*.

**Figure 4:**
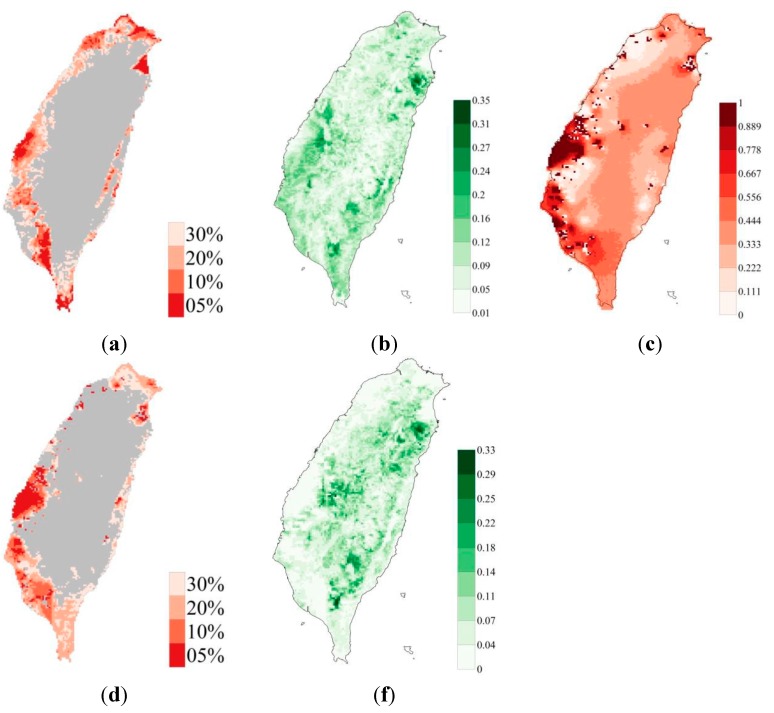
(**a**) Conservation areas with top 5%, 10%, 20% and 30% priorities, calculated by ensemble zonation; (**b**) Standard deviation of among 900 conservation priority realizations; (**c**) Spatially explicit proportion of 1000 iterations in which at least one heavy metal concentration exceeded lowest concentration threshold; (**d**) Average remediation priority of sites with top 5%, 10%, 20% and 30% conservation priorities, in terms of both ecological value and projected heavy metal concentrations, and (**e**) Standard deviation of remediation priority at sites with top 5%, 10%, 20% and 30% priorities in terms of both ecological value and projected heavy metal concentrations.

**Figure 5:**
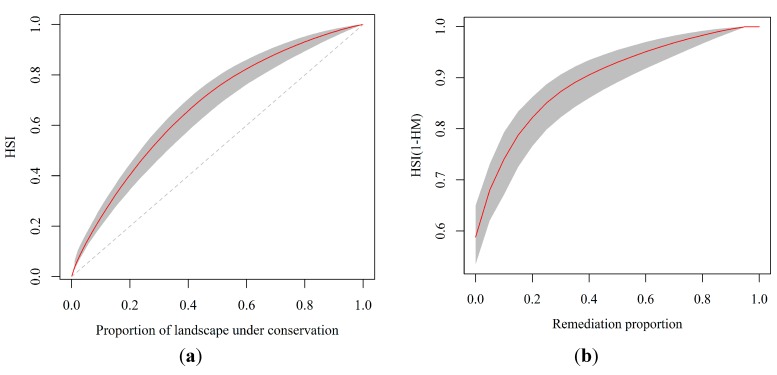
(**a**) Proportion of species’ habitat under protection as a function of total HSI value and proportion of total landscape under conservation; (**b**) Proportion of pollution-free habitat HSI(1-HM) on potentially contaminated area (grey color: 95% confidence interval). The HSI is the habitat suitability index from zero to one; HM is the heavy metal contamination: HM=1 when a site is projected as contaminated and therefore unsuitable for habitation.

Figure 3 shows the spatial distribution of eight focal species, estimated using ensemble SDMs. The mean validation AUC values of the eight ensemble species distribution models were 0.82, 0.86, 0.96, 0.93, 0.95, 0.76, 0.72 and 0.86. The areas that were selected by ensemble zonation increase as the conservation priority or representation target increases, especially in the northwestern and southwestern parts of the study area (Figure 4). The average proportions of suitable habitats for each of the eight species that were covered by the reserve areas with the top 5%, 10%, 20%, and 30% conservation priorities ranged from 0.04 to 0.32, 0.08 to 0.48, 0.15 to 0.68 and 0.23 to 0.79, respectively (Table 1). Figure 5a shows the proportions of the study landscape under conservation.

**Table 1:** Proportion of each species habitat under top 5%, 10%, 20% and 30% conservation priority areas.

Species	5%	10%	20%	30%
*A. trivirgatus*	0.04 ± 0.01	0.08 ± 0.01	0.15 ± 0.01	0.23 ± 0.02
*M. migrans*	0.11 ± 0.02	0.21 ± 0.03	0.36 ± 0.03	0.51 ± 0.02
*H. chirurgus*	0.32 ± 0.11	0.48 ± 0.15	0.68 ± 0.18	0.79 ± 0.19
*R. benghalensis*	0.15 ± 0.02	0.29 ± 0.05	0.53 ± 0.1	0.7 ± 0.14
*G. maldirarus*	0.21 ± 0.02	0.35 ± 0.01	0.62 ± 0.04	0.78 ± 0.07
*O. lettia*	0.05 ± 0.01	0.11 ± 0.01	0.22 ± 0.02	0.33 ± 0.03
*G. taewanus*	0.07 ± 0.01	0.12 ± 0.01	0.24 ± 0.02	0.38 ± 0.02
*A. cristatellu*	0.12 ± 0.01	0.23 ± 0.01	0.44 ± 0.02	0.62 ± 0.02

**Figure 6:**
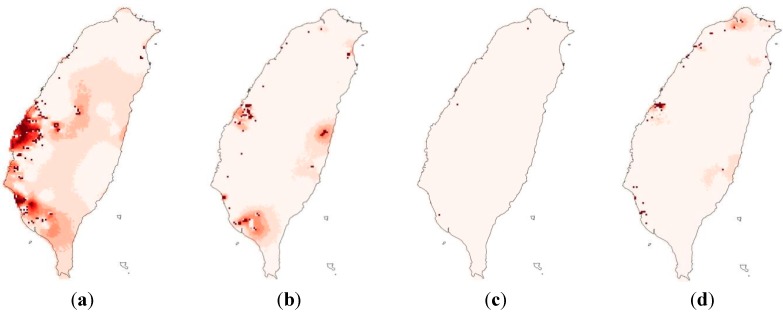

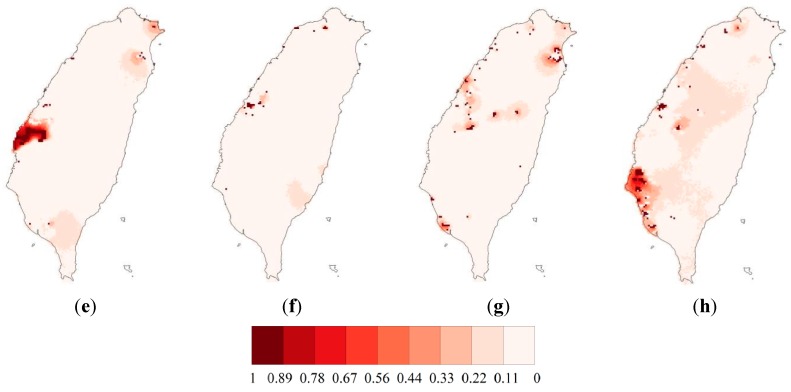
Proportion of realizations in which areas exceeded threshold concentrations of eight heavy metal soil contaminants—(**a**) As, (**b**) Cd, (**c**) Cr, (**d**) Cu, (**e**) Hg, (**f**) Ni, (**g**) Pb, (**h**) Zn—and spatial distributions of those concentrations.

### 3.2. Characteristics and Spatial Patterns of Heavy Metal Concentrations

Table S3 (in Supplementary Section 2) presents the concentrations of soil heavy metals throughout the study area. Among the eight metals, only Hg had an average concentration in the soil that exceeded the pollution threshold that was set by the EPA (Table S2), and so required intensive monitoring (class four). Table 2 presents the spatial pattern after log transformation. The datasets for the eight heavy metals in soil in Taiwan do not display any significant anisotropy (Figure S2). Therefore, isotropic semivariogram models were used to fit the spatial patterns of the log-transformed heavy metal concentrations, which are presented in Table 2.

**Table 2:** Variogram models for eight heavy metals in soil, including As, Cd, Cr, Cu, Hg, Ni, Pb and Zn (mg·kg^−1^).

Metals	Model	Nugget (C_0_)	Sill (C_0_+C)	Nugget/Sill(%) C_0_/(C_0_+C)	Range (km)	R^2^
**ln(As+1)**	Exponential	0.02	0.05	26.75	89.40	0.82
**ln(Cd+1)**	Gaussian	0.00	0.00	49.86	89.10	0.66
**ln(Cr+1)**	Linear	0.03	0.03	82.24	155.10	0.19
**ln(Cu+1)**	Spherical	0.03	0.08	43.59	52.70	0.88
**ln(Hg+1)**	Gaussian	0.00	0.04	0.25	26.80	0.57
**ln(Ni+1)**	Spherical	0.02	0.10	15.39	36.80	0.59
**ln(Pb+1)**	Spherical	0.01	0.04	31.84	117.70	0.88
**ln(Zn+1)**	Exponential	0.04	0.09	43.97	195.60	0.90

### 3.3. Simulated Heavy Metal Distribution and Identified Contaminated Areas

In the western part of the study area, As, Cd and Hg concentrations exhibit high local uncertainties (Figure 6). The local uncertainties in the concentrations of As, Cd and Zn are noticeably higher in the south-western part of the study area. Figure 4c shows the local uncertainties (in the areas that are potentially contaminated by any of the eight heavy metals. Table 3 presents the proportions of the conservation areas with the top 5%, 10%, 20% and 30% conservation priorities, and their overlap with contaminated areas associated with 0.5, 0.6, 0.7, 0.8 and 0.9 local uncertainties. The highest proportion of the total conservation area that is identified as contaminated is 42% (Table 3), and is associated with the top 5% conservation priority and a local uncertainty level of 0.5. The lowest proportion of the total conservation area that is identified as contaminated area is 14% (Table 3), and is associated with the top 30% conservation priority and a local uncertainty level of 0.9. Table S4 (see Supplementary file) presents the global uncertainty in the concentrations of heavy metals in soil.

**Table 3:** The proportions of conservation areas under top 5%, 10%, 20% and 30% conservation priority identified as contaminated areas associated with local uncertainty levels of 0.5, 0.6, 0.7, 0.8 and 0.9.

	HMLN	0.5	0.6	0.7	0.8	0.9
CP						
**5%**		0.42 ± 0.16	0.29 ± 0.15	0.24 ± 0.13	0.2 ± 0.11	0.19 ± 0.1
**10%**		0.41 ± 0.11	0.31 ± 0.12	0.25 ± 0.1	0.21 ± 0.08	0.19 ± 0.07
**20%**		0.36 ± 0.07	0.27 ± 0.08	0.21 ± 0.06	0.17 ± 0.05	0.16 ± 0.04
**30%**		0.33 ± 0.05	0.25 ± 0.05	0.18 ± 0.04	0.15 ± 0.03	0.14 ± 0.03

HMLN: local uncertainty in concentrations of multiple heavy metal pollutants. CP: conservation priority.

### 3.4. Remediation Prioritization and Robustness of Decisions

Figure 4d plots the distributions of the areas with the top 5%, 10%, 20% and 30% priorities for remediation. Figure 5b plots the increase in the value of HSI(1-HM), herein referred to as the pollution-free habitat, and the corresponding confidence interval (grey) associated with the false positive rate, corresponding to different proportions of remediated land. A method for making decisions related to remediation was used in the analysis of different ecological value requirements (EVEPI), false positive rate (FPR) tolerances and robustness requirements (Table 4). The remediation proportion of zero, as in the current situation, is the lowest required ecological value (0.48–0.72) and a false positive rate of 0. Varying SDM and soil heavy metal model outputs are such that subsequent remediation proportions correspond to varying levels of uncertainty in both the required false positive rates and the ecological value requirements.

**Table 4:** Relationship between proportion of remediation area and ecological value requirement (EVEPI) as well as varying false positive rates (FPR).

Expected Decision Efficacy		EVEPI	50%	60%	70%	80%	90%	100%
	FPR							
Robust 0.8	0%		−	1%–2%	6%–8%	−	−	−
	10%		−	−	−	15%–21%	−	−
	20%		−	−	−	−	30%–32%	−
	30%		−	−	−	−	36%–42%	−
	40%		−	−	−	−	45%–50%	−
Robust 0.9	0%		−	1%	7%–8%	−	−	−
	10%		−	−	−	16%–19%	−	−
	20%		−	−	−	−	−	−
	30%		−	−	−	−	37%–41%	−
	40%		−	−	−	−	46%–49%	−
Robust 0.95	0%		−	−	−	−	−	−
	10%		−	−	−	18%	−	−
	20%		−	−	−	−	−	−
	30%		−	−	−	−	38%–40%	−
	40%		−	−	−	−	47%–48%	−

## 4. Discussion

### 4.1. Spatial Pattern and Corresponding Uncertainty in Concentrations of Multiple Soil Heavy Metals Based on Joint- Simulation

In this study, the moderate nugget-to-sill ratios of As, Cd, Cu, Pb and Zn indicate moderate spatial autocorrelations, suggesting that both natural and anthropogenic sources contribute to their presence. In contrast, the low (C_0_/(C_0_ + C)) (<25%) [44,45] of Hg and Ni concentrations in soil suggest strong spatial dependence and intrinsic determining factors. The high (C_0_/(C_0_ + C)) (>75%) [44,45] of Cr suggests weak spatial autocorrelation and anthropogenic factors. The range represents the maximum distance at which the measured values of two samples are autocorrelated, and can also implicitly indicate similar sources. The similar ranges of As (89.4 km) and Cd (89.1 km) suggest that both originate from the same sources [46]. Despite an intermediate nugget-to-sill ratio, the range of Zn is much larger than those of other metals, at approximately 195.6 km, indicating prevalent Zn pollution.

The joint-simulation maps indicate more severe heavy-metal pollution in western Taiwan than in other areas. Moreover, the spatial uncertainty analyses, based on the joint-simulated realizations, reveal a high spatial consistency between Cr, Cu and Ni distributions, which results in high joint probabilities (low global uncertainty) [41]. In this study , when the critical proportion is 75%, the joint probabilities for Cd, Cr, Cu, Ni, Pb and Zn reach one, indicating that the areas with critical proportions of more than 75% can be used reliably to identify these heavy metals [6,41,47].

### 4.2. Ecological Hot Spots and Heavy Metal Contaminants in Soil

The discovery of heavy metal pollution in and around protected areas and ecological hotspots, as revealed by a number of studies, such as that of Fang *et al.* in 2012, is disconcerting—especially with respect to bird species that inhabit environments in which soil is contaminated by heavy metal, as such species are at increased risk of accumulating these contaminants through trophic-level interactions [1,13]. In this study, the habitats of the eight bird species of interested were concentrated in low-altitude areas, where most industrial activities take place or have taken place. As predicted, the results indicate that the areas with top conservation priority tend to be those that are most likely to be contaminated by heavy metals [7]. Moreover, many of the focal birds are high trophic level species, so they are affected more than other species by biomagnification [1,48], as heavy metals that are taken up from the soil by lower trophic organisms accumulate in the tissues of their predators [13].

### 4.3. Remediation Priority based on Ecological Conservation Priority

**Figure 7:**
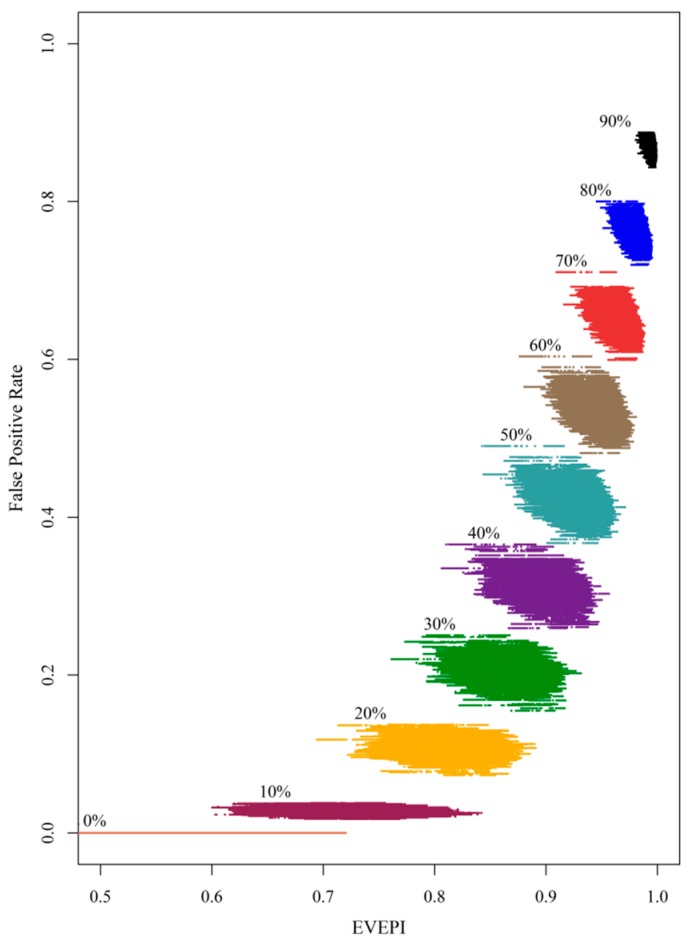
Varying total ecological value requirements associated with varying false positive rates for various remediation area proportions (0% to 90%).

The areas with the highest remediation priorities were concentrated in the western, south-western and north-eastern parts of the study area, closely corresponding to both highly polluted areas and, unfortunately, high priority conservation areas. As the remediation area increases from the current situation, zero, to 90% of polluted area the EVEPI increases drastically, particularly at the beginning of the remediation action, indicating an initial high return on investment, which is followed by diminishing returns. The confidence interval of the EVEPI proportions, indicated by the various projection outcomes that are presented in the scatter plot in Figure 7, reveals that the uncertainty of EVEPI corresponding to false positive rates vary according to different remediation goals.

A higher EVEPI corresponds to a higher false positive rate, reflecting intrinsic trade-offs between high EVEPI and low false positive rates [49,50]. In this study, the robustness of a decision is defined as the proportion of realizations which meet specific FPA and EVEPI goals, as set by decision-makers. As seen in Table 4, as the required robustness increases, acceptable remediation proportions decrease for a given ecological value (EVEPI) and required false positive rate (FPR). Decisions concerning the degree of remediation that achieves specific goals are influenced by uncertainty of measured data that arises from a lack of information [51].

## 5. Conclusions

Owing to the cost of remediation and difficulties in predicting the concentrations of heavy metal contaminants in soil, a robust decision-making process that considers the spatial uncertainty of the habitats of species and the distributions of multiple heavy metals is crucial in delineating high priority remediation areas. To address the sheer complexity of this problem, the proposed approach integrates U-WEDGE, sGs, spatial uncertainty analysis, a SDM ensemble modeling technique, a systematic conservation tool and a proposed approach for making decisions concerning remediation in lowland Taiwan. In this work, a combination of U-WEDGE and sGs was found to be the most effective approach, and successfully generated 1000 realizations of eight heavy metal concentrations over the study area. Moreover, based on the variations between realizations, a map of local uncertainty in each heavy metal distribution revealed the locations of potentially contaminated areas. Moreover, an ensemble modeling method was applied to 900 outputs of Zonation, a systematic conservation tool, derived from corresponding SDM outputs (300 outputs from three SDMs for eight protected bird species). A decision-making remediation approach that accounted for both the conservation priority of selected bird species and spatial uncertainty in multiple heavy metal distributions was used to evaluate the robustness of different remediation decisions. Finally, the effectiveness of varying levels of remediating action based on identified remediation priorities was determined in terms of ecological value requirements, expected false positive rates and the desired decision robustness.

## Supplementary Files

Supplementary File 1
