# Supplementary material for: Avian Conservation Areas as a Proxy for Contaminated Soil Remediation
Source: Int J Environ Res Public Health. 2015 Jul 17;12(7):8312–31. doi: 10.3390/ijerph120708312 (PMC4515724; doi:10.3390/ijerph120708312)
Supplement: Supplementary File 1 [file ijerph-12-08312-s001.pdf]

Supplementary Information

Avian Conservation Areas as a Proxy for Contaminated Soil Remediation

1. Information of Selected Species

The eight selected bird species inhabit agricultural areas of Taiwan. These species are locally threatened by various pressures and are protected by the Taiwanese government [1]. These eight species are not an exhaustive list of all endangered species that are affected by heavy metal contamination, but serve as an example to demonstrate the effectiveness of the remediation prioritization method for species conservation proposed in this study.

Accipiter trivirgatus is a resident raptor species that is relatively common in wooded areas and in open areas of lower elevation. It preys on rodents, birds, lizards and frogs. *Milvusmigrans* is a resident raptor species that inhabits agricultural and aquatic areas in low-lying areas of Taiwan. The population was common and widespread in Taiwan before 1970s but has experienced a great reduction of numbers since then (less than 300 individuals after 1990), due to a variety of reasons, not least of which is poisoning. It feeds on various vertebrates but mainly carcasses. *Otuslettia* is a nocturnal raptor species that is relatively common in wooded areas at lower elevations of Taiwan. It preys on insects, rodents, birds, frogs, and lizards. *Hydrophasianuschirurg* is a rare resident species that prefers ponds covered by floating plants. It once had a vast distribution and large population size in Taiwan but is currently limited to a few hundred individuals in southern Taiwan. It feeds on aquatic insects, tadpole, spiders, and snails. *Rostratulabenghalensis* is a common resident species of Taiwan and inhabits rice paddies or wetlands on plains. It feeds on insects, snails, earthworms, and seeds. *Glareolamaldivarum* is a relatively uncommon summer visitor of Taiwan and prefers partially barren lands, especially arid farmlands. It mainly feeds on aerial insects. *Garrulaxtaewanus* is a species endemic to Taiwan and is uncommon in lower elevation areas. It prefers shrubs and forages mainly on insects, fruits and seeds. *Acridotherescristatellus* is a resident species of Taiwan and prefers open areas. Its population has been greatly reduced due to inter specific competition of other invasive mynas. It prefers foraging close to the ground and feeds mainly on insects and fruits.

Table S1. Background information of selected species.

Scientific Name	Conservation Status	Population Status	Habit	Diet	Habitat
A. trivirgatus	Rare and valuable	uncommon	nonmigratory	Carnivorus	forest
M. migrans	Rare and valuable	common	nonmigratory	Carnivorus	forest
H. chirurgus	Rare and valuable	uncommon	nonmigratory	omnivorous	wetland
R. benghalensis	Rare and valuable	locally abundant	nonmigratory	Carnivorus	wetland
G. maldirarus	Rare and valuable	uncommon	migratory	Carnivorus	wetland
O. lettia	Rare and valuable	uncommon	nonmigratory	Carnivorus	forest
G. taewanus	Rare and valuable	uncommon	nonmigratory	omnivorous	forest
A. cristatellu	conservation-deserving	common	nonmigratory	omnivorous	farm and grassland

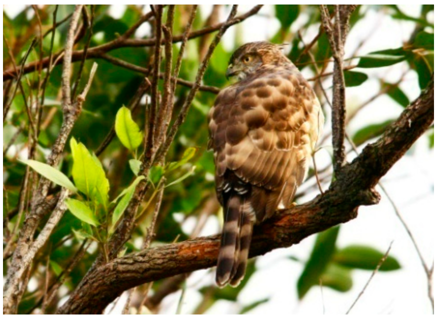
(**a**)

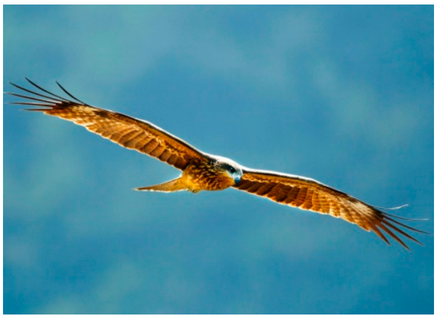
(**b**)

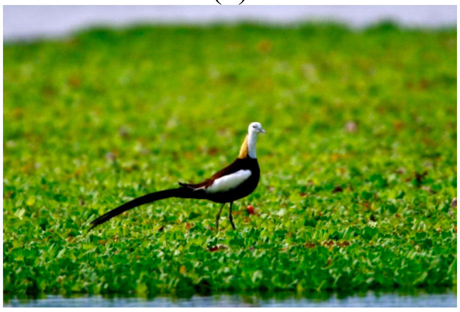
(**c**)

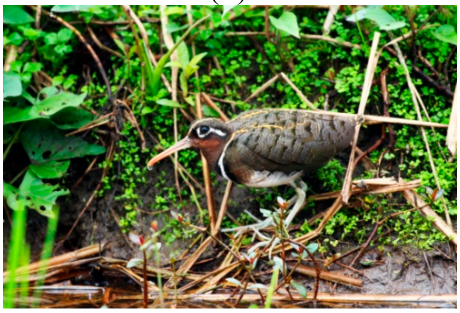
(**d**)

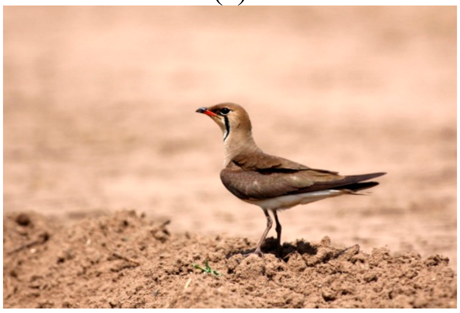
(**e**)

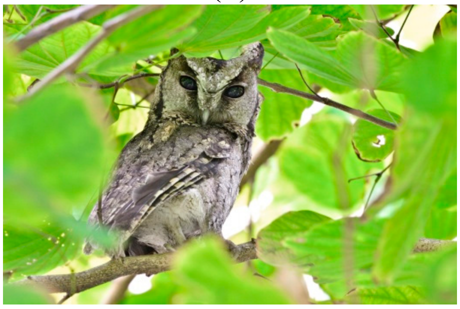
(**f**)

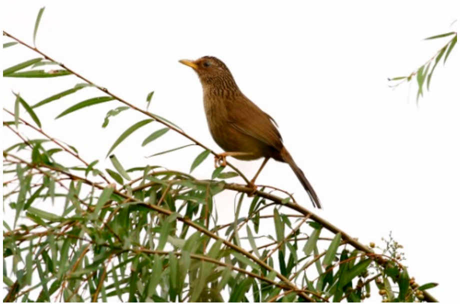
(**g**)

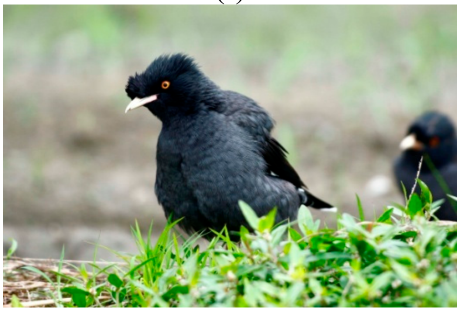
(**h**)

Figure S1. (**a**) *A. trivirgatus*; (**b**) *M. migrans*; (**c**) *H. chirurgus*; (**d**) *R. benghalensis*; (**e**) *G. maldirarus*; (**f**) *O. lettia*; (**g**) *G. Taewanus*; and (**h**) *A. cristatellu.* (Mr. Li-Pin Wang provided the above photos except for photo (**f**) which was provided by Mr. Albert Hu).

2. Background Information of Heavy Metal Samples

Sample units correlated to grid sizes of 100 ha, which was also set as the resolution cell sizes of distribution simulations. The heavy metal concentration in each 100 ha grid was calculated as the average of 30 points within each grid. A total of 2183 soil sample units were considered from all across Taiwan; except in the mountainous center of the study area, whose soil had been identified as

containing relatively low levels of heavy metal concentration early on in the survey process and was therefore not included in extensive national surveying.

Table S2. Soil heavy metal classes in Taiwan [2].

Soil heavy metals	1	2	3	4	5
**As**		<4	4–9	10–60	>60
**Cd**		<0.05	0.05–0.39	0.40–10	>10
**Cr**		<0.10	0.10–10	11–16	>16
**Cu**	<1	1–11	12–20	21–100	>100
**Hg**		<0.10	0.10–0.39	0.40–20	>20
**Ni**		<2	2–10	11–100	>100
**Pb**		<1	1–15	16–120	>120
**Zn**	<1.5	1.5–10	11–25	26–80	>80

Unit: mg/kg.

Table S3. Descriptive statistics for 8 heavy metals in soil, including As, Cd, Cr, Cu, Hg, Ni, Pb and Zn ($mg \cdot kg^{-1}$).

Metals	Mean	sd	Median	Min	Max	Skewness	Kurtosis	C.V. (%)
As	7.14	3.71	6.99	0.46	25.93	0.60	0.59	51.92
Cd	0.21	0.20	0.17	0.00	2.75	4.84	44.15	95.33
Cr	1.06	1.57	0.77	0.00	22.56	7.63	81.43	147.97
Cu	10.06	11.29	7.57	0.18	137.46	5.86	47.67	112.25
Hg	0.44	1.06	0.11	0.00	7.17	3.76	14.43	242.20
Ni	3.41	5.32	2.42	0.00	88.61	8.16	99.58	156.13
Pb	10.15	4.50	9.49	1.07	43.47	1.62	6.91	44.32
Zn	16.87	17.14	14.18	1.17	283.17	8.12	100.43	101.61

Table S4. The global uncertainty of the hotspots of the eight 8 heavy metal soil pollution, As, Cd, Cr, Cu, Hg, Ni, Pb, and Zn. Note: CP represents critical proportion.

Metals / CP	As	Cd	Cr	Cu	Hg	Ni	Pb	Zn	All
0	0.00	0.00	0.00	0.00	0.00	0.00	0.00	0.00	0.00
0.05	0.00	0.00	0.00	0.00	0.00	0.00	0.00	0.00	0.00
0.1	0.00	0.00	1.00	0.00	0.00	0.00	0.00	0.00	0.00
0.15	0.00	0.00	1.00	0.00	0.00	0.00	0.00	0.00	0.00
0.2	0.00	0.00	1.00	0.00	0.00	0.00	0.00	0.00	0.00
0.25	0.00	0.00	1.00	0.00	0.00	0.00	0.00	0.00	0.00
0.3	0.00	0.00	1.00	0.00	0.00	0.00	0.00	0.00	0.00
0.35	0.00	0.00	1.00	0.00	0.00	0.56	0.00	0.00	0.00
0.4	0.00	0.00	1.00	0.07	0.00	0.56	0.00	0.00	0.00
0.45	0.00	0.00	1.00	0.15	0.00	0.56	0.01	0.00	0.00
0.5	0.00	0.17	1.00	0.60	0.00	0.56	0.05	0.00	0.00

Table S4. *Cont.*

CP \ Metals	As	Cd	Cr	Cu	Hg	Ni	Pb	Zn	All
0.55	0.00	0.56	1.00	0.60	0.00	0.56	0.57	0.00	0.00
0.6	0.00	1.00	1.00	1.00	0.00	1.00	1.00	0.00	0.00
0.65	0.00	1.00	1.00	1.00	0.00	1.00	1.00	0.00	0.00
0.7	0.00	1.00	1.00	1.00	0.00	1.00	1.00	0.16	0.00
0.75	0.00	1.00	1.00	1.00	0.00	1.00	1.00	1.00	0.00
0.8	0.00	1.00	1.00	1.00	0.00	1.00	1.00	1.00	0.00
0.85	0.02	1.00	1.00	1.00	0.04	1.00	1.00	1.00	0.00
0.9	0.17	1.00	1.00	1.00	0.49	1.00	1.00	1.00	0.01
0.95	0.93	1.00	1.00	1.00	0.88	1.00	1.00	1.00	0.21

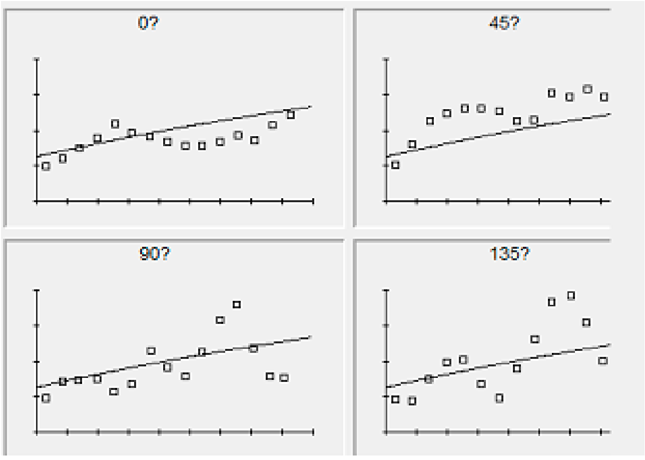

(**a**)

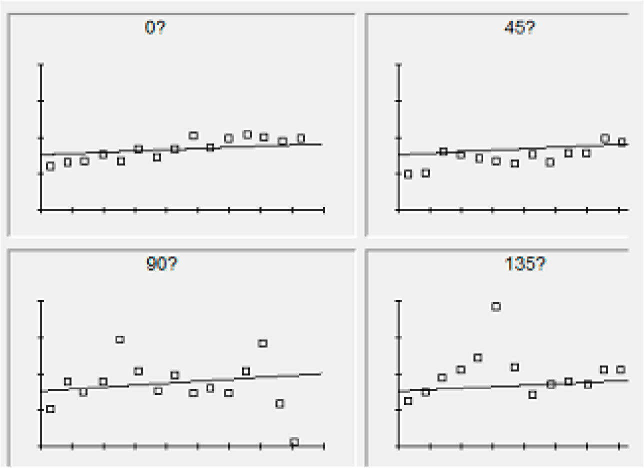

(**b**)

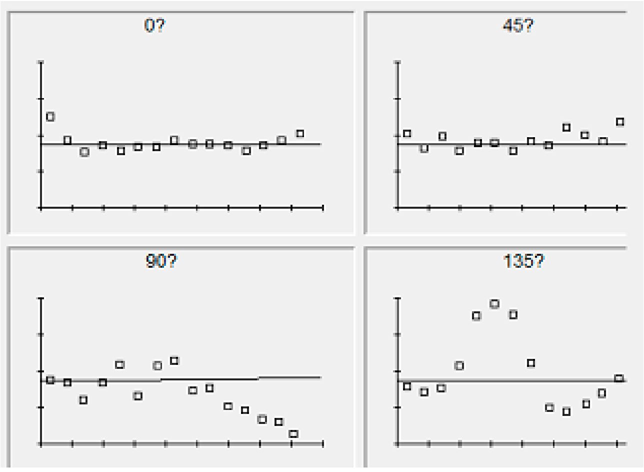

(**c**)

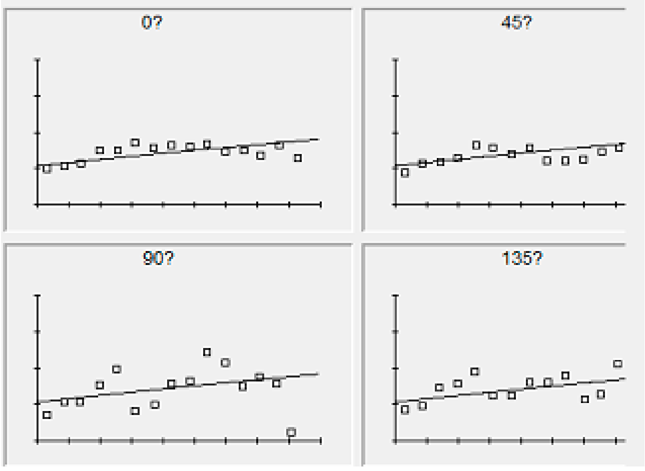

(**d**)

Figure S2. *Cont.*

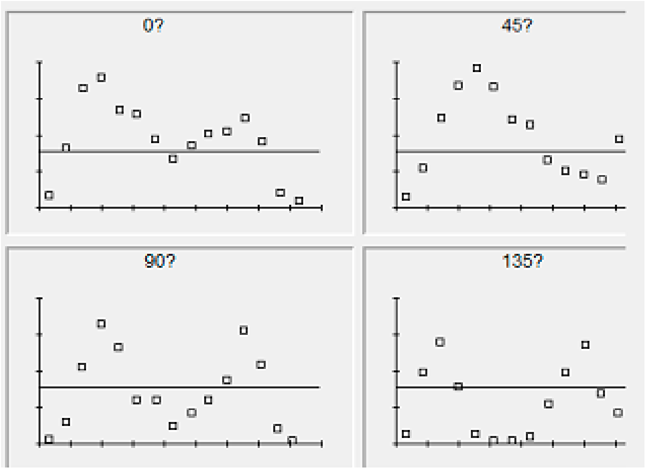

(e)

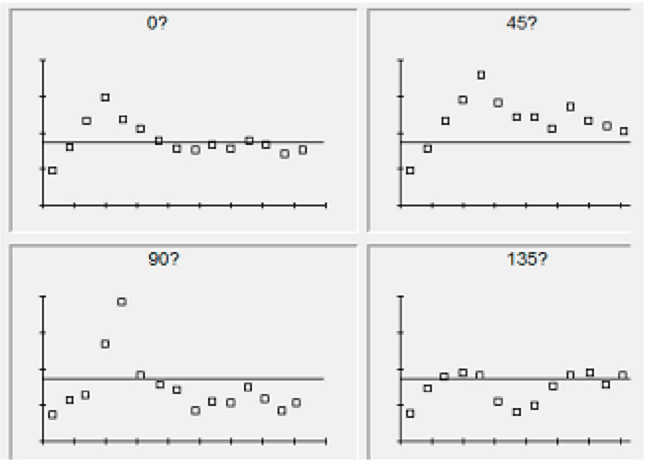

(f)

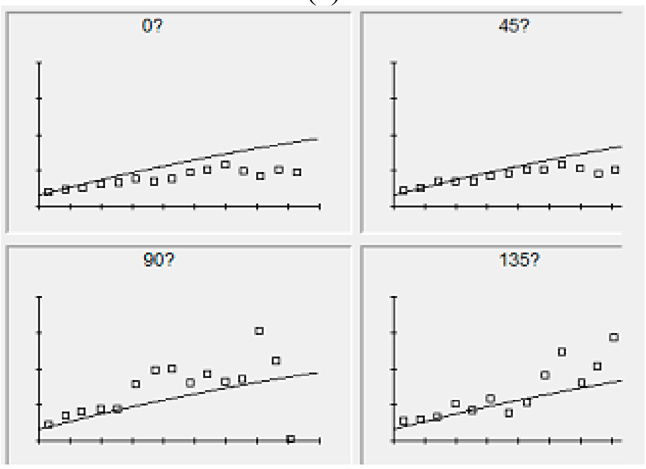

(g)

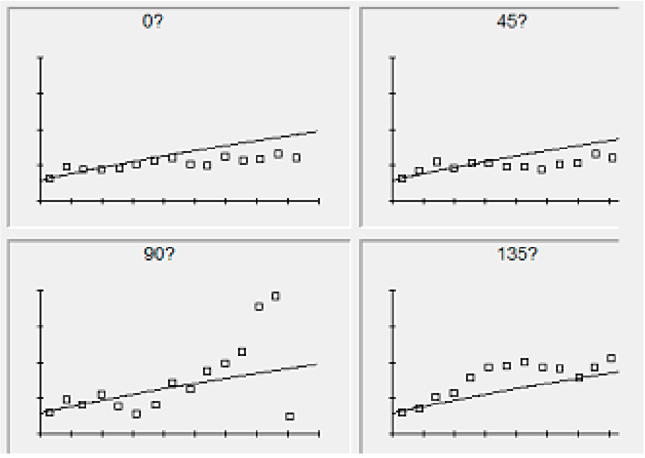

(h)

Figure S2. Anisotropic variograms for (**a**) As,(**b**) Cd, (**c**) Cr, (**d**) Cu, (**e**) Hg, (**f**) Ni, (**g**) Pb, (**h**) Zn in the direction of 0, 45, 90 and 135 degree with the distance from 0 to 160 km. Note: X axis represents distance from 0 to 160 km. Y axis represents semivariance $(\log(mg/kg + 1))^2$. All of the variograms were derived from GS+ software.

3. Species Distribution Models

3.1. General Linear Model (GLM)

The GLM provides the probability of the species presence at each location based on the driving factors considered [3]. The model quantifies the connection between species occurrences and the driving factors according to the following equation:

$$\ln\left(\frac{p_i}{1-p_i}\right) = \beta_0 + \sum_{j=1}^{k}\beta_j x_{ji}$$

and

$$p_i = \frac{\exp\left(\beta_0 + \sum_{j=1}^{k}\beta_j x_{ji}\right)}{1+\exp\left(\beta_0 + \sum_{j=1}^{k}\beta_j x_{ji}\right)}$$

where p_i is the probability of species occurrence at location i; k is the number of driving factors; x_{ji} is the driving factor j at location i; β_0 is the estimated coefficient; and β_j is the estimated coefficient corresponding to driving factor j.

3.2. General Additive Model (GAM)

The GAM provides the probability of the species presence at each location based on a nonlinear format of driving factors considered [3]. The model quantifies the connection between species occurrences and driving factors in the polynomial form given in the following equation:

$$\ln\left(\frac{p_i}{1-p_i}\right) = \beta_0 + \sum_{j=1}^{k}\beta_j f(x_{ji})$$

and

$$p_i = \frac{\exp\left(\beta_0 + \sum_{j=1}^{k}\beta_j f(x_{ji})\right)}{1+\exp\left(\beta_0 + \sum_{j=1}^{k}\beta_j f(x_{ji})\right)}$$

where p_i is the probability of species presence at location i; k is the number of driving factors; x_{ji} is the driving factor j at location i; β_0 is the estimated coefficient; and β_j is the estimated coefficient corresponding to driving factor j in the given polynomial equation.

3.3. Support Vector Machine (SVM)

Based on the driving factors x_j $(j = 1,2,\dots,k)$, SVM assigns each location i to one of two classes with corresponding labels $\mathrm{y}_i = \pm 1$ (−1: species absence; +1: species presence). The optimal separation hyperplane is defined by maximizing the margin between the training points for classes −1 and +1.
The discriminant function is defined by the following equation:

$$\mathrm{f(x)} = \boldsymbol{w}^t\boldsymbol{x} + w_0$$

where the $\boldsymbol{x}$ is a vector of k driving factors; $\boldsymbol{w}$ is the normal vector of hyperplane with k elements; w_0 is the intercept of hyperplane, which is a scalar. $\mathrm{f(x)} \geq 0$ represents species presence; In contrast, $\mathrm{f(x)} \leq 0$ represents species absence. $\boldsymbol{w}$ and w_0 were derived from minimizing the following Equation [4]:

$$\mathrm{M}(\mathbf{w}) = \frac{1}{2} \parallel \boldsymbol{w} \parallel^2 + \gamma \sum_{i=1}^{N} e_i$$

which is subjected to:

$$e_i \geq 0,$$

$$y_i(\mathbf{w}^t \boldsymbol{x}_i + w_0) \geq 1 - e_i,$$

$$\mathrm{i} = 1,2,\ldots,\mathrm{N}$$

where e_i is a slack variable of training point i; γ is a parameter to measure the amount of penalty for misclassification; N is the number of training points.

3.4. Sequential Gaussian Simulation and Normal Score Transformation

sGs is defined as a stochastic simulation, which generates a set of realizations for each cell based on the intrinsic statistical characteristics of the available data, rather than simply estimating a single output, such as a mean. The realization performed by sGs is based on the conditional distributions of the observed variable. It sequentially covers the entire surface generating values based on the known value distribution and on specific simulated values of preceding or, that is, close by cells. The assumption of the method is based on multi-Gaussian distributions for each variable. Thus, the data require a prior normal score transformation to ensure the normality of the distribution [5]. The equation of normal score transformation is as follows:

$$Y(x_\alpha) = \mathrm{G}^{-1}[\mathrm{H}^*(F(x_\alpha))], \quad \alpha = 1,\ldots,\mathrm{m}$$

where $Y(x_\alpha)$ is the transformed variable at site x_α, $\mathrm{G}^{-1}(\cdot)$ is the inverse Gaussian cumulative function (cdf), and H^* is the sample cdf of the U-WEDGE factor F.

The transformed variable $Y(x_\alpha)$ is ensured to satisfy the assumption of the Gaussian distribution so that sGs can be applied based on $Y(x_\alpha)$. When the simulation is finished, the simulated Y normal score will be back-transformed to simulate the F value [6].

4. Environmental Background Variables

Table S5. Environmental variables for species distribution models.

Biogeographic Factors	Environmental Variables	*A. trivirgatus*	*M. migrans*	*H. chirurgus*	*R. benghalensis*	*G. maldirarus*	*O. lettia*	*G. taewanus*	*A. cristatellu*
Geographic information	Presence of major city	−0.21 (<0.05)	---	−0.91 (<0.05)	---	---	−0.16 (0.168)	---	---
Ecoregion	Watershed boundary	−0.43 (<0.05)	0.76 (<0.01)	1.79 (0.052)	---	---	−0.65 (<0.01)	−0.44 (<0.05)	---
Vegetative cover	Presence of forest patch	---	---	---	−0.84 (<0.05)	−0.9 (<0.05)	---	0.39 (<0.01)	---
	Vegetation map	---	---	−1.68 (<0.01)	−0.23 (0.09)	−0.33 (<0.01)	0.21 (<0.05)	−0.15 (0.088)	−0.14 (0.109)
	Naturalness index (1–10 classes)	0.36 (0.067)	---	−7.55 (<0.05)	−1.39 (0.126)	---	---	−0.85 (<0.001)	−0.79 (<0.01)
	NDVI	0.65 (<0.001)	---	0.85 (0.068)	---	---	0.86 (<0.001)	0.2 (0.08)	−0.18 (0.146)
GIS derived variables, distance calculation	Nearest distance to 3000m and above altitudes	0.36 (<0.05)	---	5.19 (<0.01)	−1.1 (<0.01)	0.62 (<0.05)	---	---	---
	Nearest distance to major cities	−0.43 (<0.01)	---	---	---	1.11 (<0.001)	---	0.36 (<0.05)	---
	Nearest distance to river	---	---	1.48 (<0.01)	−0.37 (<0.05)	---	0.2 (0.065)	---	---
	Nearest distance to major roads of national and provincial levels	---	---	---	---	---	0.31 (0.119)	−0.46 (<0.05)	−0.58 (<0.05)
	Nearest distance to major roads of national to county levels	0.62 (<0.001)	---	---	---	2.04 (<0.01)	−0.39 (0.138)	0.68 (<0.05)	---
	Nearest distance to sea	---	−0.44 (0.056)	---	−0.58 (0.076)	0.67 (0.057)	−0.49 (<0.01)	−0.61 (<0.001)	−0.35 (<0.05)

Table S5. *Cont.*

Biogeographic Factors	Environmental Variables	*A. trivirgatus*	*M. migrans*	*H. chirurgus*	*R. benghalensis*	*G. maldirarus*	*O. lettia*	*G. taewanus*	*A. cristatellu*
Elevation	Mean elevation within $2km \times 2km$	−0.42 (0.063)	−1.85 (<0.001)	---	---	−4.27 (0.22)	−0.6 (<0.05)	−0.73 (0.103)	−2.11 (<0.01)
	Standard deviation of elevation within $2km \times 2km$	0.47 (<0.05)	---	---	---	−2.18 (<0.05)	0.57 (<0.05)	---	---
	SLOPE	---	---	---	−2.47 (<0.05)	−1.49 (0.108)	−0.47 (0.117)	---	−0.93 (<0.01)
	Relative humidity	0.21 (0.082)	−0.35 (<0.05)	−5.49 (<0.05)	−0.78 (<0.05)	---	---	0.23 (0.064)	---
River	Presence of river within $2km \times 2km$	---	---	---	---	0.41 (<0.01)	---	---	---
Precipitation	Precipitation in January	−0.61 (<0.05)	2.35 (<0.001)	−12.85 (<0.01)	−1.64 (<0.01)	---	---	0.9 (<0.001)	---
	Precipitation in February	---	---	18.99 (<0.001)	---	---	−1.43 (<0.001)	---	0.49 (<0.01)
	Precipitation in March	1.11 (<0.001)	---	---	2.63 (<0.001)	0.6 (0.143)	0.89 (<0.01)	−0.88 (<0.001)	---
	Precipitation in April	---	---	---	−2.19 (<0.001)	−1.01 (0.05)	---	0.43 (<0.05)	---
	Precipitation in May	---	---	----	−1.17 (0.083)	−1.96 (<0.01)	0.57 (<0.01)	0.85 (<0.001)	−0.74 (<0.05)
	Precipitation in June	---	0.47 (0.073)	---	1.53 (<0.01)	---	---	---	---
	Precipitation in July	---	0.25 (0.068)	---	---	−0.49 (0.111)	---	---	−0.2 (0.13)
	Precipitation in August	---	---	3.16 (<0.05)	−0.9 (<0.05)	−1.21 (<0.01)	---	---	0.71 (<0.001)
	Precipitation in September	---	0.83 (<0.01)	---	---	1.38 (<0.001)	−0.36 (0.158)	---	---
	Precipitation in October	---	---	−17.36 (<0.05)	---	---	0.65 (<0.01)	---	---
	Precipitation in November	0.98 (<0.001)	−2.04 (<0.001)	10.45 (0.151)	1.25 (<0.01)	---	---	−0.83 (<0.001)	---
	Precipitation in December	−0.58 (0.058)	---	---	---	---	---	---	---
**Temperature**	PC1 based on T01–T12	---	---	−10.14 (<0.05)	---	---	---	0.81 (0.052)	−1.43 (<0.05)
	PC2 based on T01–T12	−0.79 (<0.001)	0.63 (<0.05)	−2.53 (<0.05)	---	---	---	---	−0.51 (<0.05)
	PC3 based on T01–T12	---	---	−1.62 (<0.05)	---	---	---	−0.3 (<0.05)	−0.29 (0.097)

References

1. Severinghaus, L.L.; Ding, T.S.; Fang, W.H.; Lin, W.H.; Tsai, M.C.; & Yen, C.W. *The Avifauna of Taiwan*; Forestry Bureau, Council of Agriculture: Taipei, Taiwan, **2010**.
2. Taiwan EPA. Available online: w3.epa.gov.tw/epalaw/docfile/149008.doc (accessed on 17 July 2015).
3. Guisan, A.; Edwards, T.C., Jr.; Hastie, T. Generalized linear and generalized additive models in studies of species distributions: Setting the scene. *Ecol. Model.* **2002**, *157*, 89–100.
4. Guo, Q.; Kelly, M.; Graham, C.H. Support vector machines for predicting distribution of Sudden Oak Death in California. *Ecol. Model.* **2005**, *182*, 75–90.
5. Goovaerts, P.; Webster, R.; Dubois, J.-P. Assessing the risk of soil contamination in the Swiss Jura using indicator geostatistics. *Environ. Ecol. Stat.* **1997**, *4*, 49–64.
6. Asghari, O.; Soltni, F.; Amnieh, H.B. The comparison between sequential gaussian simulation (SGS) of Choghart ore deposit and geostatistical estimation through ordinary kriging. *Aust. J. Basic Appl. Sci.* **2009**, *3*, 330–341.

© 2015 by the authors; licensee MDPI, Basel, Switzerland. This article is an open access article distributed under the terms and conditions of the Creative Commons Attribution license (http://creativecommons.org/licenses/by/4.0/).
